# The diagnostic value of modified systemic ınflammation score in predicting post-operative outcomes of cutaneous melanoma patients who underwent ısolated limb perfusion

**DOI:** 10.1186/s12957-021-02437-6

**Published:** 2021-11-16

**Authors:** Şevket Barış Morkavuk, Serdar Çulcu, Ebru Esen, Ali Ekrem Ünal

**Affiliations:** 1 Department of Surgical Oncology, Ankara City Hospital, Ankara, Turkey; 2 Department of Surgical Oncology, Dr. Abdurrahman Yurtaslan Research and Training Hospital, Ankara, Turkey; 3 Department of Surgical Oncology, Ankara Gülhane Research and Training Hospital, Ankara, Turkey; 4 Department of Surgical Oncology, Ankara University Faculty of Medicine, Ankara, Turkey

**Keywords:** Isolated limb perfusion, Modified systemic inflammation score, Cutaneous melanoma, Lymphocyte-to-monocyte ratio, Albumin

## Abstract

**Background:**

In-transit metastasis is considered a locoregional disease in cutaneous melanoma (CM) patients. Isolated limb perfusion (ILP) is among the treatment options in selected cases. The aim of this study was to determine the success of pre- and post-perfusion mSIS values in predicting the potential complications and the prognosis of the disease by investigating the early and long-term results of mSIS values calculated before and after ILP in CM cases with in-transit metastases.

**Materials and methods:**

Patients who underwent ILP within the period from 2014 to 2020 in our department were retrospectively scanned. A total of 20 patients were found to undergo ILP. The scores obtained from modified inflammation score (mSIS) were formulated according to albumin (Alb) and lymphocyte to monocyte ratio (LMR) scores.

**Results:**

The mean follow-up time was 20.47 months. Complications requiring surgical intervention developed in three patients. According to the Wieberdink local toxicity classification, the majority (70%) of the patients were found to be grade II. Based on pre-perfusion mSIS values, 8 patients were classified as mSIS 0 while six patients were classified as mSIS 1 and 2. Based on post-perfusion mSIS values, 14 patients and one patient were classified as mSIS 2 (70%) and mSIS 0, respectively. Accordingly, univariate analysis showed that mSIS 1 and mSIS 2 were negative prognostic factors for mean survival in the pre-perfusion period (HR 0.162, 95% CI 0.036–0.729; *p* = 0.018 and HR: 0.223, 95% CI 0.049–1.019; *p* = 0.053) whereas albumin (Alb) and lymphocyte to monocyte ratio (LMR) were not independent prognostic factors for mean survival.

**Conclusion:**

The mSIS values calculated in the pre-perfusion period can give an opinion about the OS of the patients whereas post-perfusion mSIS values may predict potential surgical complications and local toxicities.

## Background

Cutaneous melanoma (CM) is a skin malignancy originating from melanocytes—cells found in the basal membrane of epidermis that determine skin color. In comparison to other skin cells, there are 1500 melanocytes per square millimeter and they undergo mitosis less than twice a year. Therefore, the incidence of cutaneous melanoma is quite low [1]. It accounts for less than 4% of all skin cancers; however, this low incidence rate is inversely proportional to prognosis. Cutaneous melanoma causes 80–85% of all skin cancer-related deaths [2]. Cancer statistics of 2020 shows that 1,806.590 new cases of cancer have been diagnosed in the United States and 100.350 of them are malignant melanoma (5.5%). While 606,520 individuals died due to cancer, the number of deaths due to malignant melanoma is 6850 [3]. It has higher mortality rates in comparison with incidence which is attributed to both, the histopathological features and the advanced stage of the disease at the time of diagnosis.

In-transit metastasis occurs in 4–11% of patients with cutaneous melanoma 13–16 months after the initial diagnosis [4]. In-transit metastasis is defined as the presence of cutaneous melanoma cells in the cutaneous and subcutaneous lymphatic ducts between the primary tumor focus and the lymph nodes where the tumor is drained [5]. In the American Joint Committee on Cancer (AJCC) guideline, in-transit metastasis is considered a locoregional disease. Based on the tumor, node, and metastasis (TNM) classification system, they are included either in stage IIIB (no lymph node metastasis) or stage IIIC (lymph node metastasis). The overall 5-year survival rate ranges from 24 to 54% compared to lymph node metastasis and the median survival is 19 months [6]. In the presence of a limited number of metastatic lesions, treatment of melanoma includes surgical resection, radiotherapy, chemotherapy, immunotherapy [interleukin-2 (IL-2), granulocyte-macrophage colony-stimulating factor (GM-CSF), ipilimumab, tremelimumab, interferon-α), and targeted therapies (BRAF inhibitors; vemurafenib, dabrafenib, and MEK inhibitor) [7]. Since it is considered a locoregional disease, isolated limb perfusion (ILP) is among the treatment options in selected cases. The ILP method was first described by Creech and Ryan in 1958 [8]. In this technique, chemotherapy doses administered at the site between the primary tumor and the draining lymph node can be 25 times higher than those used for systemic therapy, and systemic toxicity is also avoided. As in hyperthermic intraperitoneal chemotherapy (HIPEC), the efficacy of chemotherapeutic agents is improved with regional hyperthermia in the ILP technique. Complete response is achieved in 40% of patients within a period from 3 to 6 months whereas there is a partial response in 40% and no response in 20% [9]. It is a limb-sparing procedure since it can be re-performed in cases with a complete response and partial response. However, it is associated with non-negligible morbidity (73% for erythema, 17% for edema, 2% for skin necrosis and compartment syndrome, and 0.6% for limb loss) and mortality rates [10].

Virchow was the first author who reported in 1863 that systemic inflammation has an important role in cancer pathogenesis and progression [11]. Studies have shown that chronic inflammation stimulates malignant cells to increase proliferation, angiogenesis, and thus, metastasis. Serum-based inflammation parameters [e.g., platelet to lymphocyte ratio (PLR), lymphocyte to monocyte ratio (LMR), neutrophil to lymphocyte ratio (NLR)] have been shown to be prognostic factors in various cancer types [12]. Similarly, C-reactive protein (CRP), a positive acute-phase protein (APPs), and albumin (Alb), a negative APP, are known to be inflammation biomarkers. Both parameters are used in an inflammation-based scoring system known as the modified Glasgow prognostic score (mGPS). Chang et al. described a modified systemic inflammatory scoring (mSIS) system using both serum Alb and LMR. They reported that this scoring system was superior to the traditional systemic inflammation scoring system (SIS) in renal cell cancer prognosis and gave more accurate results [13].

The aim of this study was to determine the success of pre- and post-perfusion mSIS scores in predicting the potential complications and the prognosis of the disease by investigating the early and long-term results of mSIS score calculated before and after ILP in CM cases with in-transit metastases.

## Materıals and methods

### Study population

This study was conducted under approval of Ankara City Hospital ethics committee. Patients who underwent ILP within the period from 2014 to 2020 in the Ankara University Faculty of Medicine Department of Surgical Oncology were retrospectively observed. A total of 30 patients were found to undergo ILP. Two patients with a pathological diagnosis of sarcoma and eight patients with no pre-and post-ILP inflammation parameters in laboratory tests were excluded from the study. A total of 20 patients were included in the study.

### Establishment of the modified SIS (mSIS)

The scores obtained from mSIS were formulated according to Alb and LMR scores. Patients were assigned to mSIS 0 if Alb was ≥ 4.0 g/dL and LMR was ≥ 3.4, mSIS 1 if Alb was < 4.0 g/dL or LMR was 3.4, and mSIS 2 if Alb was 4.0 g/dL and LMR was 3.4 [14].

### Description of ILP technique and post-operative patient management

Computed tomography angiography (CTA) and venous Doppler ultrasonography (USG) were performed preoperatively in all patients, who would undergo ILP, to evaluate the vascular anatomy. The presence of collateral vascular formation was detected via CTA, and thus, the uncontrolled delivery of chemotherapy drug into systemic circulation was prevented. The risk of venous complications due to an existing venous thrombosis was minimized with Doppler USG. In the lower extremities, the femoral artery and vein were used for tumors located in the thigh and its distal area, and iliac artery and vein were used for tumors located in the inguinal region. Axillary artery and vein were used in upper extremity lesions. Arteriovenous structures were suspended and 5000 international units (IU) of heparin was administered as a bolus before distal and proximal clamping. After waiting for 3 min, arteriotomy and venotomy were performed. Then, the artery and vein were cannulated using a 12F catheter. Melphalan of 100 mg/kg and 50 mg/kg was titrated for the lower and upper extremities, respectively. The perfusion concentrate was calculated as 10 mg melphalan/L for the upper extremity and 12 mg melphalan/L for the lower extremity. The melphalan + fluid combination was infused into the limb at 40° for 60 min. The perfusion rate was kept between 200 mL/min and 300 mL/min to remove cytotoxic agents released from the tumor tissue. The perfusate was passed through the hemofilter for 15 min at the end of the perfusion to avoid systemic toxicity. Following the procedure, cannulae were removed and then, arteriotomy and venotomy incisions were sutured. On the first post-operative day, the perfused limbs were covered by cotton bandages to be kept warm to prevent the risk of vascular contraction. Color and heart rate were checked every 2 h. Doppler USG was utilized in suspected cases.

### Classification of surgical complications and assessment of local toxicity

Clavien-Dindo classification was used for scoring post-operative surgical complications [15]. The patients were scored from 1 to 5 according to the complications. Post-perfusion local toxicity was evaluated using the scale developed by Wieberdink et al. in 1992 [16]. Patients were evaluated and scored for local toxicity from the beginning of perfusion to the third post-operative month.

### Study design

The pre-and post-ILP mSIS scores of the patients were calculated using Alb and LMR values. The correlation of pre-and post-ILP mSIS values with post-operative surgical complications and local toxicity was evaluated. Furthermore, the association between the mSIS values and the prognosis, overall survival (OS) rates, histopathological data, and demographic data of the disease was investigated.

### Statistical analysis

Statistical analyses were performed using SPSS Statistics for Windows, Version 22.0. G-power v3.1.9.4 was used for study power analysis. Pearson’s chi-square test and Fisher's exact test were used for evaluation of nominal data of each group whereas Student’s *t* test was used for parametric data and Mann-Whitney U test for non-parametric data. One-way ANOVA, Kruskal Wallis, and post hoc multiple comparison (Bonferroni) tests were used for the analysis of multiple groups. The paired sample t-test was used to test the significance of the difference between the arithmetic means of the two dependent parametric groups whereas the significance of the difference between the arithmetic medians of the two dependent non-parametric groups was tested using the Wilcoxon signed-rank test. Univariate or multivariate death hazard ratios (HRs) were calculated with the Cox regression model. The Kaplan-Meier method was utilized for the calculation of OS. A *p* value of < 0.05 was considered statistically significant. When the Power analysis is done for the correlation analysis, the power of the study is calculated as 0.896 when the effect size conventions is taken as 0.5, the power of the study is calculated as 0.758 when the effect size conventions is taken as 0.3, and the power of the study is calculated as 0.589 when the effect size conventions is taken as 0.1.

## Results

### Patient characteristics

This study evaluated a total of 20 patients undergoing ILP due to CM. The gender distribution of the patients was homogeneous (10 males and 10 females). The mean age of all patients included in the study was 49.55 (range 16–78) years. The mean age of male and female patients were 45.70 (range 16–75) years and 53.40 (range 19–78) years, respectively. A total of 14 patients, who were being followed and treated with the diagnosis of cutaneous melanoma in an external center, were referred to our clinic for ILP since in-transit metastasis developed during this period. The anatomical origin of CM was lower extremity (70%) in 14 patients and upper extremity in six patients (30%). Regardless of the extremity, left lateralization was observed in 55% (*n* = 11) of the patients. The lesion was in the thigh in 42.9% (*n* = 6) of patients with CM originating from the lower extremity. The lesions were found to be evenly distributed in the finger and forearm area among patients with CM originating from the upper extremity. Early mortality (first 30 days) was observed in two patients. Only one patient died from a pulmonary embolism before discharge. Six patients were alive while a total of 14 patients died due to primary cancer or secondary causes during the follow-ups. The mean follow-up time was 20.47 (range 0–82.3) months. Complications requiring surgical intervention developed in three patients. According to the Wieberdink local toxicity classification, the majority (70%) of the patients were found to be grade II. There were no patients developing deep tissue damage or requiring amputation. The mean carcinoembryonic antigen (CEA) value of the patients included in the study was 1.51 ng/mL (range 0.16–3.63) and the mean alpha-fetoprotein (AFP) value was 3.09 ng/mL (range 0.92–8.22). Table 1 presents the demographic and histopathological distribution of all patients in detail.

**Table 1:** Demographic and histopathological distrubition of the patients

**Age, year, mean ± SD, range**	49.55 ± 19.06 (16–78)
**Gender:** ***n*(%)**	
Male	10 (%50)
Female	10 (%50)
**Primary/secondary case:** ***n*(%)**	
Primary	6 (%30)
Secondary	14 (%70)
**Location:** ***n*(%)**	
Upper limb	6 (%30)
Lower limb	14 (%70)
**Lateralization:** ***n*(%)**	
Left	11 (%55)
Right	9 (%45)
**Metastatic node status:** ***n*(%)**	
Absent	4 (%20)
Present	16 (%80)
**Metastatic node, number, mean ± SD, range**	1.51 ± 1.04 (0–6)
**Pre-perfusion mSIS groups:** ***n*(%)**	
mSIS 0	8 (%40)
mSIS 1	6 (%30)
mSIS 2	6 (%30)
**Post-perfusion mSIS groups:** ***n*(%)**	
mSIS 0	1 (%5)
mSIS 1	5 (%25)
mSIS 2	14 (%70)
**Clavien-Dindo classification,** ***n*(%)**	
Grade I	12 (%60)
Grade II	3 (%15)
Grade III	2 (%10)
Grade IV	2 (%10)
Grade V	1 (%5)
**Wieberdink toxicity score,** ***n*(%)**	
Grade I	3 (%15)
Grade II	14 (%70)
Grade III	3 (%15)
**Alpha fetoprotein,** ng/mL, **mean ± SD, range**	3.09 ± 2 (0.92–8.22)
**Carcinoembryonic antigen,** ng/mL, **mean ± SD, range**	1.51 ± 1.04 (0.16–3.63)
**Lenght of hospital stay, day, mean ± SD, range**	5.10 ± 1.51 (3–8)
**Early mortality**	
Absent	18 (%90)
Present	2 (%10)
* - before discharge (due to pulmonary embolism)*	*1*
*- after discharge*	*1*
**Follow-up time, month, mean ± SD, range**	20.47 ± 21.87 (0–82.3)
**Survive**	
Live	6 (%30)
Death	14 (%70)

^x^Early mortality (first 30 days): observed in two patients. One patient died from a pulmonary embolism before discharge

### Evaluation of pre- and post-perfusion mSIS parameters

Homogeneous distribution was observed in mSIS values measured before ILP. Eight patients were classified as mSIS 0 while six patients were classified as mSIS 1 and 2. Based on post-perfusion mSIS values, 14 patients and one patient were classified as mSIS 2 (70%) and mSIS 0, respectively. While the pre-perfusion mean Alb value was 3.80 g/dL (range 1.90–4.75), this value was found to be 2.95 g/dL after perfusion (range 1.74–4.00). This difference was found to be statistically significant (*p* < 0.01). There was no statistically significant difference between the pre-and post-perfusion median LMR values (3.59 vs 2.56, *p* = 0.654). The pre-perfusion mean hemoglobin (Hgb) value was 13 g/dL (range 7.90–18.60) whereas this value was calculated as 10.61 g/dL after perfusion (range 8–13.80). This decrease was statistically significant (*p* < 0.01). Similar to LMR, there was no significant difference between pre- and post-perfusion median CRP values (3.6 vs 7.27, *p* = 0.455). In other words, perfusion did not lead to a significant increase in LMR and CRP (Table 2).

**Table 2:** Pre-perfusion and post-perfusion, analysis of dependent variables

Variables	Pre-perfusion	Post-perfusion	***p*** value
**Albumin, g/dL, mean ± SD**	3.80 ± 0.69	2.95 ± 0.60	< 0.01^t^
**median (range)**	3.95 (1.9–4.75)	3.05 (1.74–4)	
**LMR, mean ± SD**	3.63 ± 1.73	4.15 ± 4.93	
**median (range)**	3.59 (1.14–6.77)	2.56(0.61–23)	− 0.654^Z^
**Hgb, g/dL, mean ± SD**	13 ± 2.47	10.61 ± 1.74	
**median (range)**	13 (7.9–18.6)	10.95(8–13.8)	< 0.01^t^
**CRP, mg/L, mean ± SD**	17.27 ± 45.23	13.04 ± 18.87	
**median (range)**	3.6(1.68–201)	7.27(1–85)	− 0.455^Z^

*t* paired samples *t* test, *Z* Wilcoxon signed-ranks test

### Comparison of pre- and post-perfusion mSIS groups with clinicopathological parameters

The evaluation of pre-perfusion mSIS groups according to gender, affected limb, lateralization, and location distribution showed no statistically significant difference. There was no significant difference in the distribution of the groups according to mean and median values of age, pre-perfusion Hgb, pre-perfusion CRP, lymph node metastasis, CEA, AFP, and hospitalization time. However, a statistically significant difference was observed in the distribution of mSIS groups according to pre-perfusion Alb value, pre-perfusion LMR value, and follow-up times. The median follow-up time of patients was 21.21 months in the pre-perfusion mSIS 0 group whereas this time was 17.61 months in mSIS 1 and 3.71 months in mSIS 2. Although this difference may seem like an inverse ratio at first glance, it will be understood to be significant when the OS rates, which are mentioned in the following chapters, are examined. Because the OS rate decreases as the mSIS value increases. This significant difference was due to the difference in the follow-up time of patients in the mSIS 0 and mSIS 2 groups (*p* = 0.034).

The evaluation of post-perfusion mSIS groups according to gender, affected limb, lateralization, and location distribution showed no statistically significant difference. There was no statistically significant difference in the distribution of the groups according to the mean and median values of the following variables: age, post-perfusion Alb, Hgb, CRP, CEA, AF, and follow-up times. On the other hand, a statistically significant difference was found in the distribution of post-perfusion mSIS groups according to lymph node metastasis, number of lymph node metastases, and hospitalization time. While 13 of 16 patients with lymph node metastases were in the post-perfusion mSIS 2 group, only one patient in the post-perfusion mSIS 0 group had lymph node metastasis (*p* = 0.035). The median hospitalization time of the patients was 6 days in mSIS 2 group, 4 days in mSIS 1, and 3 days in mSIS 0 (*p* = 0.032) (Table 3).

**Table 3:** Relationship between pre-perfusion and post-perfusion mSIS groups with clinico-pathological factors

Variables	Pre-perfusion		Post-perfusion	
	Data	***p*** value	Data	***p*** value
**Age, year, mean ± SD**				
mSIS 0	47.88 ± 21.74		78	
mSIS 1	50.50 ± 19.18	*p* = 0.954^F^	55.20 ± 23.96	*p* = 0.198 ^F^
mSIS 2	50.83 ± 18.54		45.50 ± 16.19	
**Gender:** ***n***				
**Male**				
mSIS 0	4		0	
mSIS 1	3		3	
mSIS 2	3	*p* = 1.00^χ2^	7	*p* = 0.549^χ2^
**Female**				
mSIS 0	4		1	
mSIS 1	3		2	
mSIS 2	3		7	
**Primary/secondary case:** ***n***				
**Primary**				
mSIS 0	2		0	
mSIS 1	2		2	
mSIS 2	2	*p* = 0.924^χ2^	4	*p* = 0.712^χ2^
**Secondary**				
mSIS 0	6		1	
mSIS 1	4		3	
mSIS 2	4		10	
**Location:** ***n***				
**Upper limb**				
mSIS 0	2		0	
mSIS 1	1		2	
mSIS 2	3	*p* = 0.418^χ2^	4	*p* = 0.712^χ2^
**Lower limb**				
mSIS 0	6		1	
mSIS 1	5		3	
mSIS 2	3		10	
**Lateralization:** ***n***				
**Left**				
mSIS 0	4		1	
mSIS 1	3		2	
mSIS 2	4		8	
**Right**				
mSIS 0	4		0	
mSIS 1	3		3	
mSIS 2	2	*p* = 0.790^χ2^	6	*p* = 0.522^χ2^
**Metastatic node status:** ***n***				
**Absent**				
mSIS 0	1		0	
mSIS 1	2		3	
mSIS 2	1	*p* = 0.610^χ2^	1	***p*** **= 0.035**^χ2^
**Present**				
mSIS 0	7		1	
mSIS 1	4		2	
mSIS 2	5		13	
**Metastatic node, number, mean ± SD**				
mSIS 0	2.63 ± 1.76	*p* = 0.560 ^F^	5	***p*** **= 0.05** ^F^
mSIS 1	1.83 ± 1.47		1 ± 1.41	
mSIS 2	3.88 ± 2.36		2.86 ± 1.70	
**Lymphocyte to monocyte ratio (LMR), mean ± SD**				
mSIS 0	5.05 ± 1.14	***p*** **= 0.001** ^F^	7	***p*** **= 0.015** ^H^
mSIS 1	3.39 ± 1.42		8.60 ± 8.15	
mSIS 2	1.97 ± 0.98		2.36 ± 1.85	
**Albumin, g/dL, mean ± SD**				
mSIS 0	4.33 ± 0.25	***p*** **< 0.01** ^F^	4	*p* = 0.1 ^F^
mSIS 1	3.93 ± 0.33		3.16 ± 0.64	
mSIS 2	2.96 ± 0.57		2.80 ± 0.52	
**C-reactive protein, mg/L, mean ± SD**				
mSIS 0	4.72 ± 3.84	*p* = 0.196^H^	1.5	*p* = 0.303 ^H^
mSIS 1	3.12 ± 1.42		6.48 ± 3.89	
mSIS 2	48.15 ± 78.20		16.21 ± 21.86	
**Hemoglobin, g/dL, mean ± SD**				
mSIS 0	14.06 ± 1.33	*p* = 0.058 ^F^	8.4	*p* = 0.136 ^F^
mSIS 1	13.53 ± 2.57		11.74 ± 1.32	
mSIS 2	11.06 ± 2.75		10.37 ± 1.72	
**Lenght of hospital stay, day, mean ± SD**				
mSIS 0	4.63 ± 1.50	*p* = 0.127 ^H^	3	***p*** **= 0.032** ^H^
mSIS 1	4.67 ± 1.21		4 ± 1.22	
mSIS 2	6.17 ± 1.47		5.64 ± 1.33	
**Follow-up time, month, mean ± SD**				
mSIS 0	32.89 ± 27.89	***p*** **= 0.034** ^H^	70.70	*p* = 0.314 ^H^
mSIS 1	18.62 ± 13.83		13.57 ± 9.56	
mSIS 2	5.77 ± 5.10		19.35 ± 21.38	

*χ*2 chi-square test, *F* one-way ANOVA test, *H* Kruskal-Wallis test

### Relevance of pre- and post-perfusion mSIS for surgical complications and local toxicity

The surgical complication and local toxicity rates of the pre- and post-perfusion mSIS groups were evaluated. There was no statistically significant difference in the distribution of pre-perfusion mSIS groups according to Clavien-Dindo complication classification and Wieberdink local toxicity classification (*p* = 0.188 and *p* = 0.786). Similarly, no statistically significant difference was observed in the distribution of post-perfusion mSIS groups according to surgical complication and local toxicity classifications (*p* = 0.555 and *p* = 0.110). However, in post-perfusion mSIS groups, all patients having grade III and IV complications according to the Clavien-Dindo classification were found to be in the mSIS 2 group. Similarly, 10 of 14 patients with grade II local toxicity and all three patients with grade III local toxicity according to the Wienberdink scale were also in the mSIS 2 group (Table 4).

**Table 4:** Effect of pre-perfusion and post-perfusion mSIS on surgical complications and local toxicity

	**Clavien-Dindo classification**					***p*** **value**	**Wierberdink classification**			***p*** **value**
	**Grade 1**	**Grade 2**	**Grade 3**	**Grade 4**	**Grade 5**		**Grade 1**	**Grade 2**	**Grade 3**	
**Pre-perfusion**										
mSIS 0	6	1	0	1	0	0,188^χ2^	2	5	1	0.786^χ2^
mSIS 1	4	0	0	1	1		0	5	1	
mSIS 2	2	2	2	0	0		1	4	1	
**Post-perfusion**										
mSIS 0	1	0	0	0	0	0,555^χ2^	1	0	0	0.110^χ2^
mSIS 1	4	0	0	0	1		1	4	0	
mSIS 2	7	3	2	2	0		1	10	3	
	**Clavien-Dindo classification**						**Wieberdink toxicity grading**			
**Grade I**	Any deviation from the normal post-operative course without need of intervention						No visible effect			
**Grade II**	Complication requiring pharmacological treatment						Slight erythema and/or edema			
**Grade III**	Complication requiring surgical,endoscopic, or radiological intervention						Considerable erythema and/or edema with blistering			
**Grade IV**	Life threatening complication requiring admission to intensive care unit						Extensive epidermolysis and/or obvious damage to deep tissues with a threatened or actual compartment syndrome			
**Grade V**	Death						Severe tissue damage necessitating amputation			

*χ*2 chi-square test

### The association between the early mortality and OS rates according to the pre- and post-perfusion mSIS scores

**Fig. 1:**
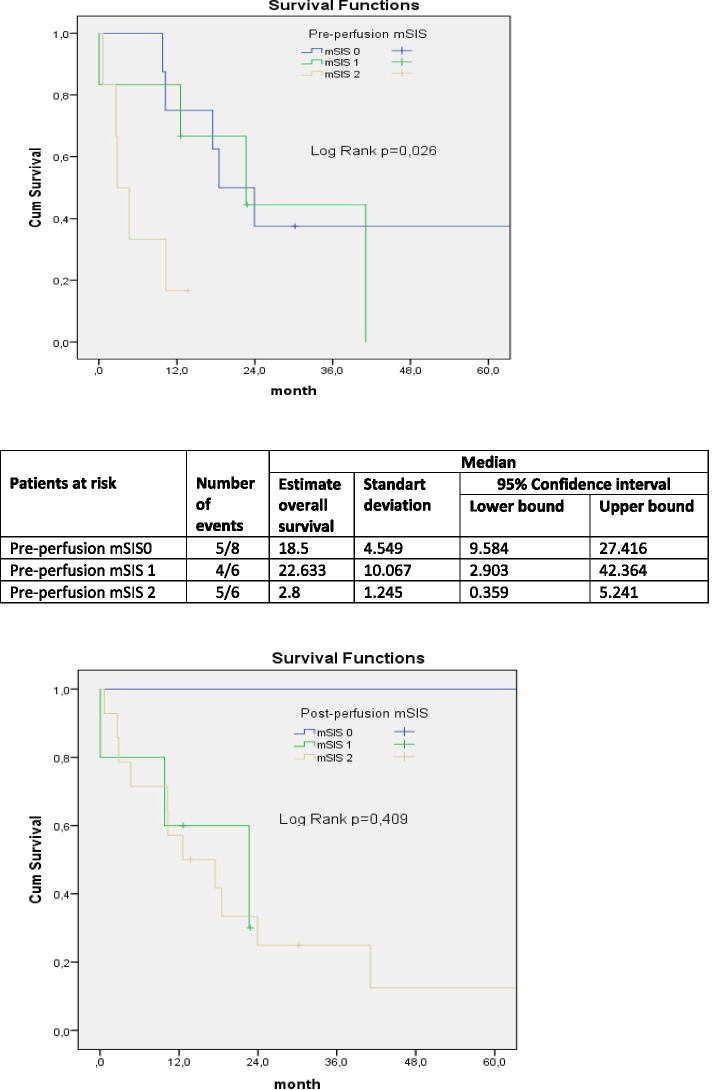
Kaplan-Meier analysis for OS according to The pre-perfusion mSIS and post-perfusion mSIS

Early mortality and OS rates of the patients, who were categorized according to their pre- and post-perfusion mSIS scores, were analyzed. There was no statistically significant difference in the distribution of pre- and post-perfusion mSIS groups according to early mortality (*p* = 0.477 and 0.672). Two patients with early mortality were both in the pre-perfusion mSIS 1–2 and post-perfusion mSIS 1–2 groups. The OS curves were generated by Kaplan-Meier analysis. There was a statistically significant correlation between the pre-perfusion mSIS groups (log-rank chi-square = 7.32, *p* = 0.026) whereas there was no statistically significant difference between the mean survival curves of the post-perfusion mSIS groups (log-rank chi-square = 1.786, *p* = 0.409) (Fig. 1). These findings were confirmed with the Cox-Regression test. Table 5 shows the univariate and multivariate survival analysis of mSIS and its components in detail. Accordingly, univariate analysis showed that mSIS 1 and mSIS 2 were negative prognostic factors for mean survival in the pre-perfusion period (HR 0.162, 95% CI 0.036–0.729; *p* = 0.018 and HR 0.223, 95% CI 0.049–1.019; *p* = 0.053) whereas Alb and LMR were not independent prognostic factors for mean survival (Table 5).

**Table 5:** Univariate and multivariate analysis of pre-perfusion and post-perfusion mSIS for overall survival

Characteristics	Univariate analysis		Multivariate analysis	
	HR (95% Cl)	***p*** value	HR (95% Cl)	***p*** value
**Pre-perfusion**				
mSIS 0				
mSIS 1	0.162 (0.036–0.729)	**0.018**	0.018 (0.00–4.023)	0.146
mSIS 2	0.223 (0.049–1.019)	0.053	0.074 (0.005–1.131)	0.061
**Pre-perfusion**				
Albumin < 4.0 g/dL	2.559 (0.870–7.526)	0.088	0.364 (0.013–10.491)	0.556
Albumin ≥ 4.0 g/dL				
**Pre-perfusion**				
LMR < 3.4				
LMR ≥ 3.4	2.027 (0.622–6.606)	0.241	0.269 (0.023–3.151)	0.296
**Post-perfusion**				
mSIS 0				
mSIS 1				
mSIS 2	0.938 (0.253–3.474)	0.924	0.933 (0.140–6.228)	0.943
**Post-perfusion**				
Albumin < 4.0 g/dL	26.558 (0.013–	0.397		
Albumin ≥ 4.0 g/dL	52393.2)			
**Post-perfusion**				
LMR < 3.4				
LMR ≥ 3.4	1.374 (0.452–4.172)	0.575	0.994 (0.200–4.927)	0.994

## Dıscussıon

Cutaneous melanoma is a rare type of skin cancer with high mortality. Its incidence has been dramatically increased in recent years. This increase is reported to be 33% for men and 23% for women [17]. Cutaneous melanoma is a malignancy with a highly poor prognosis. The loss of average life expectancy of a person diagnosed with cancer is about 16.6 years whereas this loss is 20.4 years in cutaneous melanoma [18]. Surgical treatment is performed only for palliative purposes in patients with advanced-stage cutaneous melanoma and does not contribute to survival. In-transit metastasis is considered as a locoregional disease in both National Comprehensive Cancer Network (NCCN) and AJCC guidelines and is considered to be stage III. Therefore, systemic treatment is supported with surgical interventions such as local excision or ILP, which is a routine procedure in our clinic.

Factors affecting early and long-term results of surgical intervention have been investigated in many studies and undoubtedly, serum-based inflammatory parameters and scoring systems developed based on them have recently come to the fore. This scoring system, which is called systemic inflammatory response, has been replaced by mSIS with the inclusion of new parameters over time. The present study investigated the early and long-term results of limb perfusion method according to the mSIS scoring system in cutaneous melanoma cases with in-transit metastasis. While planning this study, the patients were evaluated in two steps. The mSIS scores were calculated before and after the perfusion. Thus, the effects of the mSIS scoring system on the post-operative early and long-term outcomes of both the disease and the perfusion process were evaluated separately. The literature review has shown that there is no study investigating the effects of SIS or mSIS on the outcomes in patients with CM or patients undergoing limb perfusion. It has been further observed that there is no study focusing on predicting the outcomes of limb perfusion. All of the studies are on the contribution of perfusion to OS and disease-free survival (DFS) [10, 19]. Therefore, this is the first study in this regard as the systemic inflammatory response is utilized and is a two-step study.

We built the design of the present study on mSIS. It could have been performed only using SIS, without modification. However, we are aware of the fact that the accuracy rate of a test in which multiple parameters are used together is always higher than the univariate analysis. The best example of this is the combined use of gamma-probe and blue dye in sentinel lymph node sampling; which increases the sensitivity from 40 to 90–98%. Furthermore, Chang et al. reported in their article published in 2015 that compared to SIS, mSIS provided more accurate results in predicting prognosis in patients with renal cell carcinoma [13]. The important issue here is which factor should be added to the traditional inflammatory parameters for mSIS. While Chang et al. used Alb for modification, Huang He et al. used CRP [20]. Both of these two parameters have been recently been added to the SIS scoring system. Therefore, they have no absolute superiority over each other. We also preferred to use Alb in this study because in-transit metastases were large and ulcerated in some of the patients. This would lead to an increase in CRP levels and erroneous classification in mSIS groups. Although Alb is a nutritional indicator, it is recognized as a factor affecting the OS in various types of cancer. It has been confirmed that Alb can protect cell growth and DNA replication and exert antioxidant effects on carcinogens [21]. In many types of cancer (particularly in the gastrointestinal region) and in the presence of chronic systemic inflammation, the ability of the liver to synthesize Alb is reduced, causing hypoalbuminemia development [22]. Malnutrition may also contribute to the decrease in Alb levels, leading to the weakening of the cellular and humoral immune system and thus to errors in the anti-tumor mechanisms. However, since the rate of inadequate oral intake is very low in CM located in the limb, the risk of malnutrition is lower than in other types of cancer. In light of this data, we believe the choice of Alb is the right decision for the present study.

There is no consensus about which inflammatory parameters to use in SIS. There are studies using parameters such as lymphocyte, neutrophil, monocyte, and thrombocyte separately whereas their ratio combinations to each other have also been used in many studies [23, 24]. We preferred to use LMR in the present study. Lymphocytes are involved in anticancer immunity by preventing the proliferation, invasion, and metastasis of tumor cells. Low lymphocyte levels are responsible for insufficient immune response against tumor cells [25]. Unlike lymphocytes, monocyte contributes to the progression of tumor cells due to its transformation into the tumor-associated macrophages (TAMs) in the tumor microenvironment. Tumor-associated macrophages are responsible for tumor growth, angiogenesis, and metastasis [26]. In other words, high monocyte levels are an indicator of poor prognosis. Therefore, we used the ratio of two opposite parameters to each other. The cut-off value of this ratio was calculated based on the cut-off value determined in other mSIS studies because the present study has limited number of patients as in other studies on limb perfusion. Furthermore, the literature review has shown that the limb perfusion studies available do not use serum-based inflammatory parameters. During this selection, two different rates were observed. Cut-off value was accepted as 4.4 by Chang et al. while it was accepted as 3.4 by Lin et al. [13, 27]. In the preparation phase of the present study, when the cut-off value was accepted as 4.4, the pre-perfusion mSIS groups were observed not to be homogeneously distributed. However, when the ratio was taken as 3.4, this distribution was found to be homogeneous. Therefore, the cut-off value was accepted as 3.4 in the present study.

The correlation of pre- and post-perfusion mSIS with surgical complication and local toxicity was evaluated and no statistically significant correlation was observed between pre- and post-perfusion mSIS groups and these two types of complications. However, a detailed review of the data revealed that although there was no statistically significant correlation between the post-perfusion mSIS groups and complications, there were significant numerical differences. First of all, none of our patients had deep tissue damage, which was classified as grade IV or V based on the Wieberdink toxicity scale, and no amputation was needed. All grade II toxicities were observed in the mSIS 1 and 2 groups whereas grade III toxicity was detected only in the mSIS 2 group. There was no numerical difference in the distribution of pre-perfusion mSIS groups according to local toxicity classification. The patients were equally distributed in all three grades. Similarly, grade III, IV, and V complications, which are considered to be more severe in Clavien-Dindo complication classification, were observed to develop in the mSIS 1 and mSIS 2 groups. All grades showed equal distribution in pre-perfusion mSIS groups. We believe that it would be meaningless if pre-perfusion mSIS groups affected local toxicity and surgical complication as these complications occur after perfusion and therefore we would like to mention that the authors of the present study are highly satisfied with these findings. We do not expect the data detected in the preoperative period to affect the incidence of complications. The incidence of post-operative complications is higher in patients with malnutrition and lower Alb levels [28]. However, low Alb levels are less likely in cutaneous melanoma arising from the limbs. Furthermore, since the perfusion procedure is not an urgent intervention, it may be delayed until adequate albumin replacement is provided. In the present study, the mean pre-perfusion Alb values of the patients was 3.80. Fortunately, both types of complications were more closely associated with the post-perfusion mSIS groups because the mSIS system used in this study contained Alb, lymphocytes, and monocytes. Furthermore, as mentioned earlier, Alb is a negative acute phase reactant besides being a malnutrition indicator. Therefore, insufficient immune response may occur in the presence of low Alb levels. The authors have no doubt that the statistically insignificant but numerically significant finding will be statistically significant with a sufficient number of patients. This difference in complication rates led to a statistically significant difference in the length of stay in the post-perfusion mSIS groups (*p* = 0.032). The length of stay was longer in the post-perfusion mSIS 2 group. This outcome causes a delay in returning to daily life after surgery. Adequate albumin support and appropriate antibiotic therapy in the pre-perfusion period may decrease mSIS value. Thus, a stronger immune response will occur and complication rates will decrease. Moreover, the decrease in complication rates will shorten the length of stay and allow patients to return to their daily lives earlier. This will ensure that the socio-economic losses of the patients are minimum.

Finally, the effects of pre-and post-perfusion mSIS groups on OS were investigated. In contrast to local toxicity and surgical complications, both numerically and statistically significant correlations were found. There was no statistically significant difference between the OS curves of the post-perfusion mSIS groups whereas there was a significant correlation between the OS curves of the pre-perfusion mSIS groups (0.409–0.026). As it is considered meaningless that local toxicity and complications are affected by the data in the pre-perfusion period, we would also consider it meaningless that the OS, a highly important outcome, was affected by post-perfusion data. It is unacceptable that Alb and hematological parameters that change after an hour of perfusion affect the life span of a person. In the study by Chang et al., who first introduced the mSIS concept, and in other similar mSIS studies, the data of the patients were analyzed based on the laboratory results obtained in the preoperative period [13, 14]. The OS rates of the pre-perfusion mSIS 1 and mSIS 2 groups were found to be lower than the mSIS 0 group. We also correlated this difference with univariate analysis. While HR was statistically significant in the mSIS 1 group, it was very close to statistical significance level in the mSIS 2 group (0.018–0.053). Similarly, Chang et al. and Lin et al. reported that OS decreased as mSIS increased in patients with renal cell cancer and stomach cancer, respectively [13, 27]. Compatible with their study, we attribute this result to increased monocyte levels and decreased lymphocyte and Alb levels as mSIS values increased. As stated before, high monocyte is responsible for metastasis and angiogenesis, while low Alb and lymphocyte are responsible for insufficient immune response. Therefore, early metastasis and recurrence are observed in patients with high mSIS, which contributes negatively to the prognosis and survival.

This study has several limitations. Firstly, randomization could not be done because of its retrospective design and it may have been subject to selection bias. Furthermore, several patients were excluded as their data were not able to be reached. This caused a limitation in the number of patients. Secondly, limb perfusion is not a routine surgical procedure. As in HIPEC, it is not a method yet accepted by surgeons and oncologists. Although our clinic is one of the few experienced centers in Turkey in this regard, our patient population is not sufficient. The fact that patients with an indication for extremity perfusion are not referred to our clinic is another reason for the limited number of patients. For these reasons, our sample size could not reach the sufficient number of patients. In the analysis using *t* test for study power, 95% accuracy rate and when effect size conventions 0.5 was selected, the minimum sample size was 34 patients for correlation analysis and 184 patients according to Wilcoxon and Mann-Whitney U test. However, as we have seen in other studies, single-center studies on ILP have low patient numbers. Therefore, multicenter studies rather than a single center are needed for ILP.

## Conclusıon

In conclusion, the mSIS values calculated in the pre-perfusion period can give an opinion about the OS of the patients whereas post-perfusion mSIS values may predict potential surgical complications and local toxicities. Sufficient Alb replacement and appropriate antibiotic therapy after limb perfusion may prevent the development of potential complications. Thus, patients can return to their daily lives earlier and socio-economic losses may be kept to a minimum.

## Data Availability

The datasets used and/or analyzed during the current study are available from the corresponding author on reasonable request.

## Abbreviations

mSIS: Modified systemic inflammatory scoring
CM: Cutaneous melanoma
NLR: Neutrophil to lymphocyte ratio
LMR: Lymphocyte to monocyte ratio
CRP: C-reactive protein
Alb: Albumin
ILP: Isolated limb perfusion
HIPEC: Hyperthermic intraperitoneal chemotherapy
OS: Overall survival
AJCC: American Joint Committee on Cancer
NCCN: National Comprehensive Cancer Network
APPs: Acute-phase protein
